# Prediction of antimicrobial susceptibility of pneumococci based on whole-genome sequencing data: a direct comparison of two genomic tools to conventional antimicrobial susceptibility testing

**DOI:** 10.1128/jcm.01079-24

**Published:** 2024-12-31

**Authors:** Gerardo J. Sanchez, Lize Cuypers, Lies Laenen, Peter Májek, Katrien Lagrou, Stefanie Desmet

**Affiliations:** 1 Laboratory of Clinical Microbiology, KU Leuven, Department of Microbiology, Immunology and Transplantation 573654 , Leuven, Flanders, Belgium; 2 Department of Laboratory Medicine, National Reference Centre for Invasive Pneumococci, University Hospitals Leuven 60182 , Leuven, Flanders, Belgium; 3 Ares Genetics, OpGen 693982 , Vienna, Austria; 4 Day Zero Diagnostics Inc., Watertown, Massachusetts, USA; National Institute of Allergy and Infectious Diseases, Bethesda, Maryland, USA

**Keywords:** whole-genome sequencing, antibiotic susceptibility test, genomic, prediction

## Abstract

Determination of antimicrobial resistance (AMR) in pneumococcal isolates is important for surveillance purposes and in a clinical context. Antimicrobial susceptibility testing (AST) of pneumococci is complicated by the need for exact minimal inhibitory concentrations (MICs) of beta-lactam antibiotics. Two next-generation sequencing (NGS) analysis tools have implemented the prediction of AMR in their analysis workflow, including the prediction of MICs: Pathogenwatch (https://pathogen.watch/) and AREScloud (OpGen). The performance of these tools in comparison to phenotypic AST following EUCAST guidelines is unknown. A total of 538 *Streptococcus pneumoniae* isolates were used to compare both tools with phenotypic AST for penicillin, amoxicillin, cefotaxime/ceftriaxone, erythromycin, trimethoprim-sulfamethoxazole, and tetracycline. Disk diffusion was performed for all isolates, and broth microdilution was performed for isolates with reduced beta-lactam susceptibility. Demultiplexed FASTQ files from Illumina sequencing, covering the whole genome of pneumococci, were used as input for the NGS tools. Categorical agreement (CA), major error (ME), and very major error (VME) rates were calculated. For beta-lactam antibiotics, CA was high (>94%) associated with none or only one ME and VME (<1%). For erythromycin and tetracycline, CA was >93% for predictions by AREScloud, while for Pathogenwatch, this ranged around 88%. For trimethoprim-sulfamethoxazole, CA was for both tools <86%. High VME rates were observed for erythromycin and tetracycline, higher for Pathogenwatch (53.6% and 47.0%, respectively) compared to AREScloud (14.3% and 19.1%, respectively). Both tools performed excellently despite the complexity of predicting beta-lactam resistance in pneumococci. Further optimization and validation are needed for non-beta-lactams since high (very) major error rates were observed.

## INTRODUCTION

*Streptococcus pneumonia* (*Spn*) is one of the leading bacterial pathogens worldwide responsible for an estimated 829,000 deaths in 2019 (1). *Spn* can cause non-invasive and invasive disease, primarily in children <5 years old and elderly populations (>65 years old). Although pneumococcal conjugate vaccines prevent *Spn* infection, pneumococcal morbidity and mortality remain significant concerns (2, 3).

The first-choice antibiotics to treat pneumococcal infections are beta-lactam antibiotics, but in Europe, about 16% of invasive pneumococci are penicillin non-wild type (4, 5). The resistance rates to other antibiotics exhibit variability with resistance rate of 17.9% (6, 7) and 16% for macrolides and tetracycline, respectively (8), while resistance is rare or absent for fluoroquinolones (1.1%) (4) and absent for glycopeptides (9).

Beta-lactam resistance in *Spn* depends on mutations in the penicillin-binding protein (PBP) genes that reduce or eliminate the affinity of the PBPs for beta-lactam drugs. *Spn’s* most important PBP genes are *pbp1a, pbp2b*, and *pbp2x* (10). It has been shown that a sole mutation in one PBP will not grant a resistance profile, and in many cases, mutations must appear concomitantly in different PBPs. This makes it more complex to predict the *Spn* resistance profile for beta-lactam antibiotics based on genetic information compared to predicting resistance to other antibiotic classes used to treat pneumococcal infections. For example, for macrolides, resistance is most often due to a target modification (*erm(B*)) or an efflux pump mechanism (encoded by the *mefA* gene). The presence or absence of a gene or a mutation in a gene itself is already an indication of resistance to the respective antibiotic.

Accurate antimicrobial susceptibility testing (AST) is essential for the targeted treatment of pneumococcal infections. Clinical microbiology laboratories use phenotypic AST methods to determine a minimal inhibitory concentration (MIC) or a zone diameter, which can be interpreted as a categorical result (S, I, or R) that indicates the chance of treatment success.

Implementing genomics and its tools in clinical microbiology laboratories and national reference centers (NRCs) is a forthcoming step toward better surveillance of AMR (11, 12). In the context of surveillance of infectious diseases, increasingly more NRCs move from conventional phenotypic and molecular characterization of isolates (e.g., serotyping, AST) to whole-genome sequencing (WGS). The advantage is that, in theory, the different characteristics of the organism can be deduced based on the whole genome instead of using multiple and often costly conventional tests. As with any other application, WGS also presents some challenges: it needs isolation of pure colonies, an acceptable sequencing depth and coverage across the genome, and the required downstream bioinformatic analyses can be complex. In addition, predicting antimicrobial resistance may be challenging, resulting in the need for phenotypic AST next to the prediction based on WGS (13).

Prediction of antimicrobial resistance based on WGS data can be performed using algorithms to identify the presence or absence of genes conferring or associated with drug resistance or mutations in these genes. Currently, more than 20 computational tools are available for antimicrobial resistance profiling (14) that can be used for a wide range of bacteria. These tools primarily identify the presence or absence of genes, also known as the rule-based approach. Among these tools, Resfinder (15) and ARIBA are the most known, and more recently, VAMPr (16), which is one of the few tools that has on top of a rules-based also a machine-learning approach (16). Unfortunately, for pneumococci, it is not only important to have information about the presence or absence of resistance-associated genes but also information about the chance of treatment success with the antibiotic. The interpretation of beta-lactam AST results of pneumococci is primarily based on MIC values. None of the aforementioned tools provide MIC information for beta-lactam antibiotics as output, hampering their use in both reference laboratories and clinical laboratories. Given the complexity of predicting *Spn* beta-lactam resistance due to altered PBPs, more pneumococcal-specific tools have been developed in recent years.

At the Centers for Disease Control and Prevention (CDC), an AMR prediction tool that associates MICs with specific *pbp* profiles, using random forest as the machine-learning algorithm, was developed (17). Later, it was validated to deal with uncharacterized PBP profiles, achieving high categorical (CA) and essential agreement (EA) rates (>93%) and low error rates (<1.4%) (18). However, as the tool was developed and validated using isolates collected in the United States, its performance on sequences of isolates from other geographical locations is not known. This AMR prediction tool has been implemented in an online freely available platform developed by the Big Data Institute and Oxford University: Pathogenwatch (PW) (19).

Another machine-learning algorithm to predict antimicrobial susceptibility, named XGBoost, is used by the tool AREScloud (20). In the latter approach, the focus is not only on the regions where resistance-associated genes are located but also on evaluating the whole genome to identify hotspots according to a given phenotype. AREScloud has been tested on 620 clinical isolates originating from respiratory tract samples, including *Staphylococcus aureus*, Enterobacterales, *Pseudomonas aeruginosa, Stenotrophomonas maltophilia*, and *Acinetobacter baumannii* (21). For the 129 species-antibiotic pairs, overall CA was 89%, with CA even exceeding 90% in 85% of pairs. Major error (ME) and very major error (VME) rates of 8% and 19%, respectively, were observed (21). In another performance study of AREScloud, antimicrobial resistance across 30 antimicrobial agents was predicted for nine different bacteria, and the tool achieved average CA, ME, and VME rates of 85.8%, 11.0%, and 21.8%, respectively (22). At present, no published evaluations on the performance of AREScloud for AST prediction of *Spn* are available.

In this study, we assessed the performance of two promising online tools, PW and AREScloud, for predicting the antimicrobial susceptibility of *S. pneumoniae* using the phenotypical AST method as a reference.

## MATERIALS AND METHODS

### Bacterial isolates

We included 538 unique clinical *Streptococcus pneumoniae* isolates from patients with invasive pneumococcal disease (IPD) between 2007 and 2022 from the Belgian reference center for invasive pneumococci. All isolates were collected from patients with IPD from 2007 to 2022. Additionally, we included 1 ATCC isolate (ATCC49619) and the 10 isolates from the EUCAST (European Committee on Antimicrobial Susceptibility Testing)/CCUG (Culture Collection University of Gothenburg) *Streptococcus pneumoniae* panel as quality control isolates as these strains have defined MIC ranges (23). We selected clinical isolates with complete disk diffusion results with variable phenotypic susceptibility profiles for different antibiotic classes to reflect true resistance rates (5). All isolates were stored at −80°C on BHI + 10% glycerol. Bacteria were subcultured on a blood agar plate and incubated overnight at 37°C in an atmosphere with 5% CO_2_ to perform AST and DNA extraction prior to sequencing.

### Phenotypic characterization

AST was performed by disk diffusion on Mueller-Hinton fastidious agar (MH-F) using disks (Beckton Dickinson, USA) for the antibiotics oxacillin, erythromycin, tetracycline, trimethoprim/sulfamethoxazole, and levofloxacin according to EUCAST guidelines (24). The oxacillin disk was used to screen for decreased beta-lactam susceptibility (25). When the corresponding oxacillin diameter was <20mm and for a random subset of 20 isolates where oxacillin diameter was >20mm, MICs were determined. Broth microdilution was performed, making use of customized Sensititre plates (see Table S1, BELKUL1, Thermo Scientific), including 11 antibiotics: amoxicillin, cefotaxime, chloramphenicol, clindamycin, erythromycin, levofloxacin, moxifloxacin, penicillin, tetracycline, trimethoprim/sulfamethoxazole, and vancomycin. In brief, colonies were added to 5mL of Mueller Hinton broth (Thermo Scientific, cat. T3462-10), until 0.5 McFarland was reached at the nephelometer. Then, 100µL of this solution was transferred to 11mL of Mueller Hinton broth with lysed horse blood (Thermo Scientific, cat. CP114-10). Using the AIM Sensititre (Thermo Scientific), 100µL was added to each well of the BELKUL1 plate. After 18–24 h of incubation at 37°C, each plate was read using the Vizion instrument (Thermo Scientific). All obtained MICs were interpreted according to EUCAST breakpoints v.13 (24).

### Genotypic characterization

DNA extraction from the pneumococcal isolates was performed with the QIAsymphony SP Instrument (QIAGEN, USA) using the DSP DNA Mini Kit with the gram-negative bacteria pre-treatment protocol. Libraries were prepared with the Nextera XT DNA Library Preparation Kit (Illumina, USA), followed by sequencing using the MiSeq instrument (version 2 or 3 kit) or HiSeq2500 with version 2 reagents (Illumina, USA). We used the demultiplexed raw FASTQ files as input for two bioinformatic tools for the prediction of antimicrobial resistance: PW (https://pathogen.watch/, Big Data Institute, University of Oxford, UK) and AREScloud (Ares Genetics GmbH, Vienna, Austria, who recently filed a petition for bankruptcy protection, with acquirement of its assets by bioMérieux SA). This tool was accessible through the link https://www.ares-genetics.cloud/ during the study period and last accessed in August 2024 but unavailable at the time of publication. Results were downloaded from each tool as .csv files.

PW is a web-based application built for genomic surveillance and epidemiology of pathogens, including *Streptococcus pneumoniae* (26). For *S. pneumoniae,* AMR prediction consists of two different parts, with the first one focusing exclusively on the prediction of MICs of beta-lactam antibiotics based on PBP profiles as described by Li et al. (18). The alleles from the PBPs (p*bp1A*, *pbp2B,* and *pbp2X*) are coded by a machine-learning approach, and the MICs are estimated for six beta-lactam antibiotics: penicillin, amoxicillin, meropenem, cefotaxime, ceftriaxone, and cefuroxime. On top of that, a categorization into S, I, and R is reported based on the estimated MIC values using CLSI guidelines from 2013 (27). The second part is based on PAARSNP, a PW module that uses BLAST against a custom library to identify the presence or absence of specific genes involved in antimicrobial resistance to infer antibiotic resistance. Based on the PAARSNP library 1313 version 0.0.16 (https://github.com/pathogenwatch/amr-libraries/blob/main/1313.toml), PW displays a results table that includes inferred resistance for 10 non-beta-lactam antibiotics: chloramphenicol, clindamycin, erythromycin, fluoroquinolones, kanamycin, linezolid, tetracycline, trimethoprim, sulfamethoxazole, and trimethoprim-sulfamethoxazole. Results were reported as “none” when no genes associated with AMR were found or as “resistant” when an AMR determinant was found. “Intermediate” results were only reported for trimethoprim-sulfamethoxazole. When resistance is inferred, the results table also displays the genes associated with the resistance as “known determinants.”

The WGS-AST models of AREScloud are machine-learning models trained on ARESdb (21), which contains WGS and AST data from proprietary and public sources. The predictive models are trained on complex feature matrices generated from WGS data. The generated features include DNA k-mers, amino acid k-mers, protein counts, protein lengths, multi-locus sequence types (MLSTs), the presence of AMR markers, DNA assembly quality control metrics, and mutation severance predictions (22). In this study, AREScloud v1.8 was used. For benzylpenicillin, amoxicillin, cefotaxime, erythromycin, and tetracycline, a beta-version of new predictive models specifically developed for *Spn* was used to predict respective MIC values. For trimethoprim-sulfamethoxazole, the custom prediction of AREScloud was used.

All analyses were performed during July–August 2023. Quality control requirements as used by the prediction tools to proceed to an analysis are detailed in Table S2.

### Categorical agreement for all antibiotics

Phenotypic AST was defined as the reference method. Among the tested isolates, disk diffusion was the reference method, and when oxacillin diameter ≥20mm—in line with the EUCAST guidelines for these isolates—penicillin, amoxicillin, and cefotaxime were deduced as susceptible (S). When the oxacillin diameter was <20mm, broth microdilution was performed and used as the reference method.

We categorized the MIC results and zone diameters (both from reference and prediction tools) into susceptibility categories: susceptible (S), susceptible increased exposure (I), or resistant (R), making use of the non-meningitis EUCAST v13.0 breakpoints (24). CA, major error (ME) rate, and very major error (VME) rate were calculated according to CLSI guidelines for verification of commercial AST systems (28) for all antibiotics with phenotypic results and available prediction for both tools (Table 1, highlighted in gray). Antibiotics without phenotypic results for all isolates (chloramphenicol and clindamycin) were excluded. Despite the intended purpose of this CLSI guideline for commercial AST system verification, we decided to use this guideline also for the prediction tools as it is a minimal criterion for AST methods to be used in a clinical diagnostic context.

**TABLE 1:** Overview of the format of antibiotic results obtained with the different AST methods^*c*^

Antibiotic	Antibiotic class	Phenotypic	Pathogenwatch	AREScloud
(Benzyl)penicillin	Beta-lactams	DD/MIC^*a*^	MIC	MIC
Amoxicillin	Beta-lactams	MIC	MIC	MIC
Cefotaxime/ceftriaxone	Beta-lactams	MIC	MIC	MIC
Cefuroxime	Beta-lactams	NA	MIC	NA
Meropenem	Beta-lactams	MIC	MIC	NA
Kanamycin	Aminoglycosides	NA	R or non-R	NA
Chloramphenicol	Chloramphenicols	MIC	R or non-R	S or R
Levofloxacin	Fluoroquinolones	DD/MIC	R or non-R^*b*^	NA
Moxifloxacin	Fluoroquinolones	MIC	R or non-R^*b*^	NA
Vancomycin	Glycopeptides	MIC	NA	NA
Clindamycin	Macrolides	MIC	R or non-R	MIC
Erythromycin	Macrolides	DD/MIC	R or non-R	MIC
Linezolid	Oxazolidinones	NA	R or non-R	NA
Trimethoprim-sulfamethoxazole	Sulfonamides	DD/MIC	R, I, or non-R	S or R
Tetracycline	Tetracyclines	DD/MIC	R or non-R	MIC

^*a*^ Oxacillin screen result in case of disk diffusion; MIC, minimum inhibitory concentration; DD, disk diffusion; R, resistant.

^*b*^ Only indication of fluoroquinolone resistance. NA, not available.

^*c*^ Highlighted in gray are the antibiotics that were included in this study.

### Essential agreement for beta-lactam antibiotics

For the isolates and antibiotics with MIC results for both prediction tools (penicillin, amoxicillin, and cefotaxime) and phenotypic MICs, EA was determined according to CLSI guidelines (28) by calculating the proportion of MIC results per antibiotic that agrees (defined as MIC within one doubling dilution of the reference MIC) with the phenotypic MIC result. Bias was calculated according to the ISO 20776-1 (29).

### Evaluation of the performance of PW and AREScloud to predict AST results

We used the acceptance criteria proposed by CLSI to evaluate the performance of the two prediction tools compared to the phenotypic AST method: >90% for CA and EA and <3% for ME and VME (28).

### Quality control of the used methods

The MIC results of CCUG and ATCC isolates obtained by phenotypic testing and prediction tools were compared with the intended results (Table S3) (23, 30). Results needed to be equal to the intended result or one dilution higher/lower to be judged as in agreement. If the predictive tool predicted no MIC for an antibiotic, the predicted categorical result needed to agree with the categorical result based on the intended MIC.

### Analysis of discordant resistance predictions by NGS tools and the phenotypic AST method

Isolates for which AMR prediction by the NGS tools disagreed with the phenotypic result were analyzed in more detail. Features that determined the AMR prediction (MIC or S/I/R category) were obtained from AREScloud, being mostly nucleotide or amino acid k-mers. These k-mers were compared to the sequence information in the library of genes used by PW for AMR prediction. To evaluate whether a resistance gene was present, we used the assemblies downloaded from the corresponding tool and used this file to perform a local BLAST, using as query the *tetM* gene (NCBI accession number FR671418—nucleotide positions 17031–18950).

### Processing time measurement

We used a randomly selected subset of 20 sequences (total size of 8,08Gb) out of the final data set of 517 sequences. The same subset was tested at three different time slots of the day for each prediction tool. Each time, a different uploading order was used to calculate the average runtime for each tool to perform AST predictions.

## RESULTS

### Phenotypic antibiotic susceptibility characterization of Spn isolates

Out of the 549 isolates (538 clinical and 11 quality control isolates), 32 isolates were excluded from further analysis due to incomplete results or complete failure. For 22 out of 32 isolates, no results were obtained by AREScloud. For 10 other isolates, incomplete results were obtained by PW since beta-lactam MIC values could not be predicted, with for one isolate also no results were obtained by AREScloud. For all 32 isolates, key quality criteria were not met. The performance analysis was conducted on 517 isolates, with a median number of reads of 807,568 (range: 121,748–3,221,586) per isolate and a median sequencing depth of 104x (range: 18–428x).

For 75.05% of isolates (*n* = 388/517), the reference method was disk diffusion, while for the remaining 24.95% (*n* = 129/517), it was broth microdilution. Table 2 summarizes the phenotypic AST results, including resistance rates for penicillin, amoxicillin, cefotaxime, and non-beta-lactam antibiotics.

**TABLE 2:** Results of phenotypical AST testing of 517 *Streptococcus pneumoniae* isolates

Antibiotic	Phenotypic characterization		
	S % (*n*)	I *% (n*)	R *% (n*)
(Benzyl)penicillin	79.1 (*409*)	16.8 (*87*)	4.1 (21)
Amoxicillin	93.6 (*484*)	1.6 (8)	4.8 (25)
Cefotaxime/ceftriaxone	94.7 (*490*)	4.3 (22)	1.0 (5)
Erythromycin	78.3 (*405*)	0	21.7 (*112*)
Trimethoprim-sulfamethoxazole	77.3 (*400*)	7.4 (*38*)	15.3 (*79*)
Tetracycline	77.7 (*402*)	0	22.3 (*115*)

### Evaluation of quality control isolates

The results obtained by the phenotypic methods and AREScloud agreed with the intended results of the 11 quality control isolates (1 ATCC and 10 CCUG isolates) for all antibiotics (Table S3). However, two results obtained by PW were discordant. Isolate CCUG-74416 was predicted as erythromycin S, while the intended result was R (MIC> 2mg/L), also indicated by AREScloud with k-mers mapping to the *erm(B*) resistance gene. Isolate CCUG-74423 was predicted as S for tetracycline by PW, while the intended result was R (MIC>8mg/L) . The R result for tetracycline was confirmed by AREScloud by k-mers mapping to the *tetM gene,* which is known to be involved in AMR. Results obtained by the phenotypic method—in this case broth microdilution—were all in agreement with the intended results.

### Performance of PW and AREScloud for beta-lactam antibiotics

Both tools had a CA exceeding 94% for penicillin (94.2%), amoxicillin (97.3%), and cefotaxime/ceftriaxone (98.5%). No ME and VME were detected, except for amoxicillin due to one disagreement between AREScloud and the phenotypic method. EA was >90% for all beta-lactam antibiotics, except for the penicillin prediction of AREScloud, which was 88.4% (see Table 3). This lower EA was mainly due to an underestimation of the penicillin MIC value of more than one dilution compared to the reference (see Table S4) in 14 isolates, resulting in another categorical result (S/I/R) for only 10 isolates. Regarding the penicillin prediction, not only a bias toward lower MIC prediction was observed for AREScloud (−51%), but also for PW, a bias (−34%) was observed, resulting in lower MICs compared to the reference MIC.

**TABLE 3:** CA, ME, VME, and EA rates for AREScloud and PW WGS-AST compared to phenotypic AST^*a*^

	AREScloud				Pathogenwatch			
Antibiotic	CA (%)	ME (%)	VME (%)	EA (%)	CA (%)	ME (%)	VME (%)	EA (%)
(Benzyl)penicillin	**94.2**	**0.0**	**0.0**	88.4	**94.4**	**0.0**	**0.0**	**92.2**
Amoxicillin	**97.3**	**0.2**	**0.0**	**95.3**	**96.5**	**0.0**	**0.0**	**96.1**
Cefotaxime/ceftriaxone	**98.5**	**0.0**	**0.0**	**97.7**	**98.8**	**0.0**	**0.0**	**98.4**
Erythromycin	**95.9**	**0.5**	14.3	NA	88.2	**0.3**	53.6	NA
Trimethoprim-sulfamethoxazole	85.5	8.8	**2.5**	NA	81.2	**1.3**	**1.3**	NA
Tetracycline	**93.8**	**2.0**	19.1	NA	88.4	**1.5**	47.0	NA

^*a*^ CA was calculated for antibiotics with complete data for phenotypical methods and predictive tools. EA was calculated only for beta-lactam antibiotics with MIC results from broth microdilution and the predictive tool. Values in bold passed the CLSI (M52 guideline) (28) acceptance criteria for AST systems: CA and EA ≥90%, ME and VME <3%. NA, not available.

### Performance of AREScloud and PW for non-beta lactam antibiotics

The CA for predictions by AREScloud was >90% for erythromycin (95.9%) and tetracycline (93.8%), while it was lower for trimethoprim-sulfamethoxazole (85.5%). None of the predictions by PW met the CLSI criteria. (see Table 3).

Both prediction tools had a low ME rate (<1%) for erythromycin but exceeded the acceptable VME rate (<3%). A VME rate of 14.3% for erythromycin was observed for AREScloud due to 16 disagreements and 53.6% for PW due to 60 disagreements. Notably, the 16 isolates that were phenotypically resistant were predicted as S by AREScloud and were also predicted as susceptible by PW. Among the additional 44 of the 60 isolates predicted “incorrectly” as S by PW (Table S5), AREScloud predicted for all these isolates MIC values >1mg/L.

For trimethoprim-sulfamethoxazole, ME rates were too high for AREScloud (8.8%) which was due to 35 isolates predicted as R while phenotypically susceptible. The VME rate met the CLSI criteria (<3%) for both tools (Table 3).

For tetracycline, both tools met the CLSI criteria for ME rates, while VME rates were too high (i.e., 19.1% for AREScloud due to 22 disagreements and 47.0% for PW due to 54 disagreements). The 22 phenotypically resistant isolates predicted as S by AREScloud and PW were characterized with a disk diffusion diameter ranging between 15 and 24mm and, therefore, called as resistant by the phenotypic method. In contrast, the additional 32 isolates predicted as S by PW were predicted as R by AREScloud with MIC values ≥4mg/L.

### Analysis of discordances of erythromycin and tetracycline prediction compared to phenotypic characterization

A more detailed analysis was performed for the 44 phenotypically erythromycin-resistant isolates, which were concomitantly predicted as resistant by AREScloud but reported as non-resistant by PW. The presence of the *ermB* gene (NCBI accession number JN899585) with >98% identity was confirmed in 36 of the 44 isolates (81.8%), while the complete *ermB* gene could not be detected in the other eight isolates. The prediction as erythromycin-resistant from AREScloud for all 44 isolates was based on the presence of two determining features, being two nucleotide k-mers mapping 100% to the *erm(B*) gene coding for erythromycin resistance.

The same approach was applied for the 32 isolates determined tetracycline resistant by the phenotypic method and AREScloud prediction while reported as non-resistant by PW. The most important feature for resistance, as identified by AREScloud to predict these 32 isolates as being resistant to tetracycline, was an amino acid k-mer (GKSLEALELEQEESIR), which mapped 100% to the translated *tetM* gene. Scanning the assemblies as obtained from PW, for the presence of the *tetM* gene (1,920bp), revealed that for 22 out of 32 isolates, the *tetM* gene was covered by two non-overlapping contigs, each representing <50% of the gene. In 10/32 isolates, the gene was covered by one contig, however, with its length corresponding to <80% of the *tetM* gene. By contrast, for the 61 tetracycline-resistant isolates for which the phenotypic method and the prediction tools agreed, the *tetM* gene was found in one contig with a coverage >93%.

### Processing time measurement

Both tools had a comparable uploading speed (PW: 33.71 Mb/s; AREScloud: 29.15 Mb/s) with an average of 02:31 for AREScloud and 3:06 for PW to provide AST results (Table S6).

## DISCUSSION

In this study, 517 *Streptococcus pneumoniae* isolates were used to assess the performance of online antimicrobial susceptibility prediction tools PW and AREScloud. For both tools, the CA, ME, and VME rates for beta-lactam antibiotics (penicillin, amoxicillin, and cefotaxime/ceftriaxone) met the criteria as proposed by CLSI (28). The EA rates met the criteria for all antibiotics with both tools (>90%), except for penicillin susceptibility prediction with AREScloud (88.4%). For the non-beta-lactams (erythromycin, trimethoprim-sulfamethoxazole, and tetracycline), the CA rates met the criteria only for erythromycin and tetracycline prediction by AREScloud. ME rates exceeded the criterion of 3% only for trimethoprim-sulfamethoxazole prediction by AREScloud, while VME rates did not meet the criteria for both erythromycin and tetracycline for both tools.

The CA, ME, and VME rates obtained with both tools for the beta-lactam antibiotics in our study are similar to the rates reported during the validation of the CDC prediction tool (18), also all fulfilling CLSI criteria. Only the EA rate of penicillin calculated for the AREScloud prediction was lower than the criterion of 90% (88.4%) and especially lower when compared to the rate obtained in the CDC validation study (>93%) (18). AREScloud used 10.493 sequences to train the penicillin model; 3,940 sequences (37.5%) correspond to the CDC training set. The additional sequences belong to public and private sources, which might have included AST results from methods other than broth microdilution. The use of results from other sources, and potentially also from different phenotypic AST methods (e.g., gradient diffusion) to train the models of AREScloud and PW, may have resulted in the prediction of lower penicillin MICs compared to MICs determined by broth microdilution making use of Sensititre. It is well studied that, especially for penicillin, certain gradient diffusion tests and commercial systems may underestimate penicillin MICs compared to broth microdilution (31). The variability in performance of penicillin MIC testing methods for *S. pneumoniae* may, thus, be an explanation of the observed bias for the prediction tools, as they are trained based on phenotypic data.

We hypothesize that the high VME rate (53.6%) observed for PW for erythromycin and the incorrect erythromycin result for one of the CCUG isolates may be due to a suboptimal detection of the *erm(B*) gene. We inferred this based on identifying the complete *ermB* gene in 36 of the 44 isolates that were categorized as non-resistant by PW. While further investigation is needed, the failure to detect erythromycin resistance might be the result of a technical bias in the process of analyzing the NGS reads from these isolates, the assembly of the contigs, or the presence of an additional macrolide resistance mechanism in these isolates not yet identified.

The high VME rate (47.0%) of PW for tetracycline may be due to a suboptimal detection of the *tetM* genes coding for tetracycline resistance. PW uses a criterion of 90% for coverage (which equals to 1,728 bp) for the detection of the *tetM* gene. Therefore, we hypothesize that the main reason that PW did not correctly identify the *tetM* gene in the context of prediction of resistance to tetracycline was that for the 32 discordant isolates, the 90% threshold for coverage was not met, either due to one contig or due to two non-overlapping contigs spanning less than 90% of the length of *tetM,* supported by all concordant isolates that did meet this criterion.

The lowest CA rates were observed for trimethoprim-sulfamethoxazole. For AREScloud, these low CA rates did not agree with previously reported high CA rates (>90%) for several other bacterial pathogens (21, 22). A possible explanation may be the difference in clinical breakpoints for trimethoprim-sulfamethoxazole between CLSI and EUCAST. Phenotypical results were determined following EUCAST guidelines, but AREScloud and PW predictions are based on CLSI breakpoints. In addition, a high ME rate was observed for AREScloud. This may be due to the AREScloud models that are not trained for trimethoprim-sulfamethoxazole to predict MIC values and can only provide a categorical result of “R” or “S” based on the CLSI breakpoints. Therefore, we assume that phenotypically “I” isolates (1–2 mg/L) might be predicted as “resistant,” while these MIC values result in S or I categorization following EUCAST guidelines. Based on our study, predictions were most accurate for antibiotics for which the tools predicted MICs [e.g., beta-lactam antibiotics (both tools) and erythromycin and tetracycline (AREScloud)] compared to antibiotics for which only a categorical result (S/I/R) was predicted.

Both prediction tools had similar processing times and provided an accessible graphical/web user interface platform that could be used without extended bioinformatic knowledge. We observed a higher rate of samples with incomplete or no results with AREScloud (4.0%) compared to PW (1.8%), which is important to consider if these tools would be used in a real-world setting. Besides the prediction of AST, both tools provide information for MLST that helps characterize pneumococcal isolates in more detail. In addition, the PW tool also reports details on the pneumococcal serotype, Global Pneumococcal Sequence Cluster (GPSC) assignment, core genome clustering, and plasmid Inc types, which are highly valuable in analyzing genomics data. Our study delivers important information about the performance of the AMR prediction tools included in PW.

This is the first study assessing the performance of two AMR prediction tools for *Spn*, including the prediction of MIC values for beta-lactam antibiotics. We included quality control isolates to monitor the performance of the tools. We evaluated clinical isolates with variable resistance profiles, representing a broad MIC range for the different antibiotics, mimicking clinical practice, and making it useful to the broad network of reference laboratories in other European countries. Considering that the genomic predictive tools might be implemented in a clinical setting in the near future, using the phenotypic susceptibility tests like disk diffusion and broth microdilution as the gold standard is a strength. This study, however, also has some limitations as we did not have MIC values for all the isolates included in our study. However, we anticipate that influence would be minimal, given that we used EUCAST-recommended protocols for disk diffusion, and quality controls met the expected criteria. The interpretation of phenotypic susceptibility testing results by EUCAST breakpoints may have impacted the evaluation of the performance of the predictive tools for predictions not based on MICs and only reported as categorical results. It is important to highlight that only for trimethoprim-sulfamethoxazole (both PW and AREScloud), erythromycin, and tetracycline (only PW), the resistance prediction was based on a training set where phenotypic AST results were interpreted using CLSI breakpoints. This divergence in interpretation guidelines can be considered a layer of complexity in evaluating tool performance. In addition, we took the phenotypic results as the gold standard and did not perform retesting in case of discordances. But we expect retesting would not change the final conclusions as quality control of our phenotypic AST methods did not detect major technical issues. We only evaluated the prediction of antibiotics that were predicted by both tools, requiring the need to evaluate other (less commonly used) antibiotics.

Overall, our results indicate that both PW and AREScloud met the performance criteria for beta-lactam antibiotics. Also, the predictions based on MICs of non-beta-lactams almost all met the CLSI criteria for implementation validation of conventional AST methods in clinical laboratories. Our findings support the idea that it is possible to predict phenotypic AST results based on WGS data of pneumococci using tools specifically designed to predict MICs. In the context of more and more pneumococcal genomes that have become available, these tools may help study large data sets to better understand (evolutions in) antimicrobial susceptibility. Additionally, these genomic tools present another option alongside phenotypic AST. This is valuable in a clinical setting and especially relevant as NRCs transition from phenotypic and PCR-based testing to WGS. Despite the promising results, some improvements on predictions of resistance of non-beta lactam antibiotics are needed.

## Data Availability

The authors confirm that all supporting data and protocols have been provided within the article or through supplementary data files. Raw sequence files have been deposited in the National Center for Biotechnology Information Short Read Archive under BioProject accession numbers PRJNA780376, PRJNA1138924, and PRJNA1144854.

## SUPPLEMENTAL MATERIAL

Supplemental tables jcm.01079-24-s0001.docx Tables S1 to S6.

ASM does not own the copyrights to Supplemental Material that may be linked to, or accessed through, an article. The authors have granted ASM a non-exclusive, world-wide license to publish the Supplemental Material files. Please contact the corresponding author directly for reuse.
