# Supplementary material for: Prediction of antimicrobial susceptibility of pneumococci based on whole-genome sequencing data: a direct comparison of two genomic tools to conventional antimicrobial susceptibility testing
Source: J Clin Microbiol. 2024 Dec 31;63(2):e01079-24. doi: 10.1128/jcm.01079-24 (PMC11837510; doi:10.1128/jcm.01079-24)
Supplement: Supplemental tables — Tables S1 to S6. [file jcm.01079-24-s0001.docx]

Supplementary materials

**Prediction of antimicrobial susceptibility of pneumococci based on whole genome sequencing data: a direct comparison of two genomic tools to conventional antimicrobial susceptibility testing**

Sanchez Gerardo J^1^, Cuypers Lize^1,2^, Laenen, Lies^1,2^, Májek, Peter^3,4^, Lagrou Katrien ^1,2^, Desmet Stefanie^1,2^#

Table S1: Sensititre plate format- BELKUL1 (Thermo Scientific)


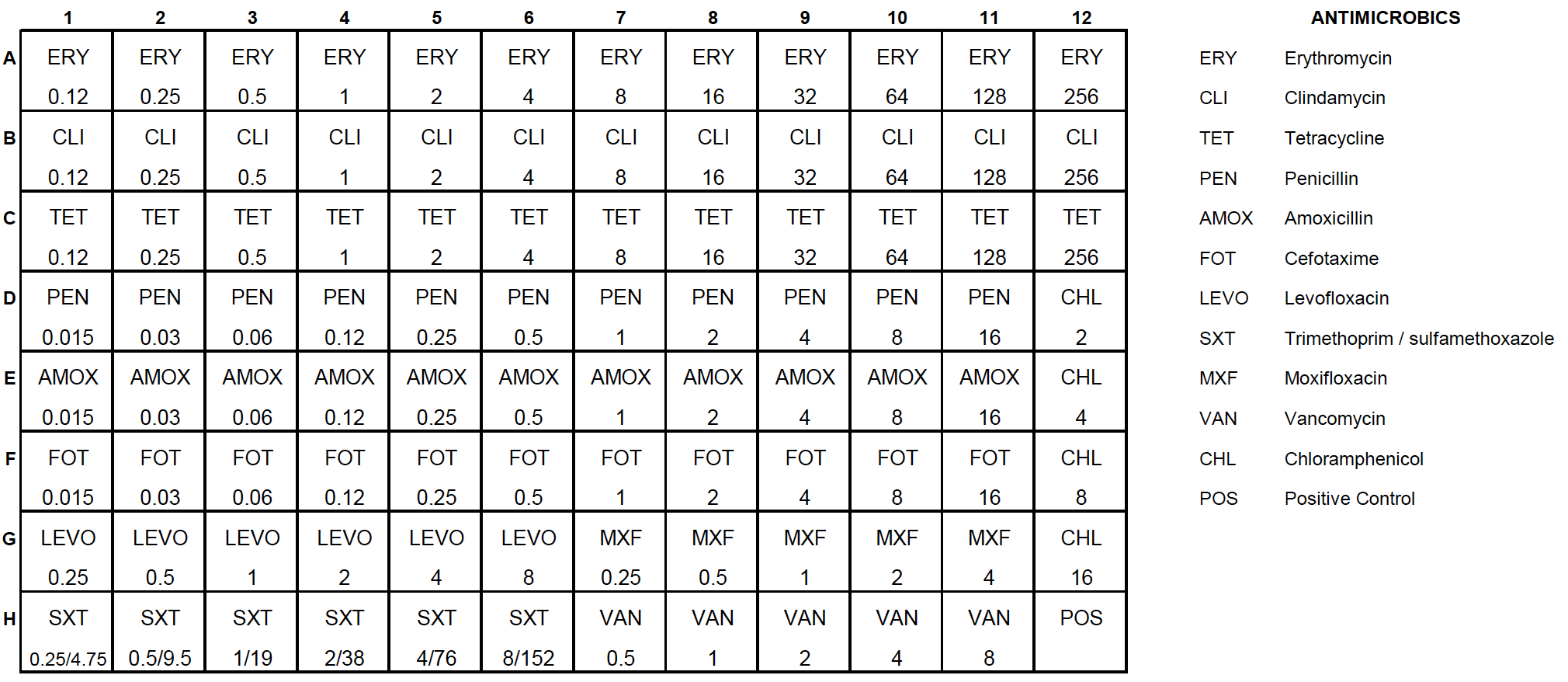


Table S2: Quality control steps in Pathogenwatch (PW) and AREScloud tool. NA: not applicable.

|  |  | **PW** | | **AREScloud** |
| --- | --- | --- | --- | --- |
|  | **Purpose** | **Fails if** | **Warning** | **No further analysis** |
| [**Fastqc**](https://www.bioinformatics.babraham.ac.uk/projects/fastqc/) | Quality control check of raw sequence data | any of the these modules fails:  Per base sequence quality,  Per sequence quality scores,  Sequence Length Distribution,  Overrepresented sequences,  Adapter Content | Warning in any module | Used but no publicly available criteria |
| [**ConFindr**](https://olc-bioinformatics.github.io/ConFindr/) | Detection of inter- and intra-species contamination | >5% of non-pneumococcal species is detected | NA | NA |
| [**Quast**](https://github.com/ablab/quast) | Quality verification of the assembly | >150 contigs or if  N50 <50000 bp | >75 contigs or if N50 <100000bp | N50 <5000 bp or L50 >500 bp or average genome size >1.5 x 2.1Mb |
| [**BUSCO**](https://busco.ezlab.org/) | Evaluation of genomic data completeness and redundancy | NA | NA | <70% completeness or >7% duplicates |

Table S3: Results for both CCUG and ATCC strains of phenotypic AST, AREScloud and PW prediction.

Agreement of results for beta-lactam and non-beta lactam antibiotics. Highlighted cells in orange are in disagreement with the intended results of the CCUG isolate.

|  | penicillin (S ≤0.06, R>2) | | | | | amoxicillin (S ≤0.5, R>1) | | | | | cefotaxime (S ≤0.05, R>2) | | | | |
| --- | --- | --- | --- | --- | --- | --- | --- | --- | --- | --- | --- | --- | --- | --- | --- |
|  | phenotypic-MIC | AREScloud-MIC | PW- MIC | CCUG -MIC range | agreement | phenotypic-MIC | AREScloud-MIC | PW- MIC | CCUG -MIC range | agreement | phenotypic-MIC | AREScloud-MIC | PW- MIC | CCUG -MIC range | agreement |
| CCUG 74415 | 0.25 | 0.50 | = 0.5 | 0.50 | Yes | 0.50 | 0.50 | = 0.5 | 0.50 | Yes | 1.00 | 0.50 | = 0.5 | 1.00 | Yes |
| CCUG 74416 | 0.06 | 0.06 | = 0.06 | 0.03-0.06 | Yes | 0.06 | 0.06 | = 0.06 | 0.06 | Yes | 0.25 | 0.25 | = 0.25 | 0.25 | Yes |
| CCUG 74417 | 4.00 | 8.00 | = 4 | 4.00 | Yes | 8.00 | 8.00 | = 8 | 8.00 | Yes | 2.00 | 1.00 | = 2 | 2.00 | Yes |
| CCUG 74418 | 4.00 | 4.00 | = 2 | 2.00 | Yes | 4.00 | 2.00 | = 2 | 4.00 | Yes | 2.00 | 4.00 | = 1 | 2.00 | Yes |
| CCUG 74419 | 4.00 | 8.00 | = 4 | 4.00 | Yes | 8.00 | 8.00 | = 8 | 8.00 | Yes | 2.00 | 1.00 | = 2 | 2.00 | Yes |
| CCUG 74421 | 2.00 | 2.00 | = 2 | 2.00 | Yes | 1.00 | 1.00 | = 2 | 2.00 | Yes | 0.50 | 1.00 | = 1 | 0.5-1 | Yes |
| CCUG 74422 | 0.12 | 0.12 | = 0.25 | 0.13 | Yes | 0.12 | 0.12 | = 0.06 | 0.06 | Yes | 0.06 | 0.12 | = 0.12 | 0.06-0.125 | Yes |
| CCUG 74423 | 0.50 | 0.25 | = 0.25 | 0.125-0.25 | Yes | 0.50 | 0.25 | = 0.25 | 0.25 | Yes | 0.25 | 0.25 | = 0.25 | 0.125-0.25 | Yes |
| CCUG 74424 | 1.00 | 0.50 | = 0.5 | 0.5-1 | Yes | 0.25 | 0.12 | = 0.12 | 0.13 | Yes | 0.25 | 0.25 | = 0.12 | 0.125-0.25 | Yes |
| CCUG 74425 | 0.25 | 0.12 | = 0.12 | 0.13 | Yes | 0.25 | 0.25 | = 0.12 | 0.125-0.25 | Yes | 0.25 | 0.25 | = 0.12 | 0.25 | Yes |
| ATCC49619 | 0.5 | 0.25 | =0.5 | 0.25-1 | Yes | 0.12 | 0.12 | = 0.12 | 0.03-0.125 | Yes | 0.12 | 0.12 | =0.12 | 0.03-0.012 | Yes |

|  | erythromycin (S ≤0.25, R>0.25) | | | | | | | | trimethoprim-sulfamethoxazole (S ≤1, R>2) | | | | | | | tetracycline (S <1, R>1) | | | | | | | |
| --- | --- | --- | --- | --- | --- | --- | --- | --- | --- | --- | --- | --- | --- | --- | --- | --- | --- | --- | --- | --- | --- | --- | --- |
|  | phenotypic- | | AREScloud | | PW- category* | CCUG | | agreement | phenotypic | | AREScloud-category* | PW- category* | CCUG | | agreement | phenotypic | | AREScloud | | PW- category* | CCUG | | agreement |
|  | MIC | category | MIC | category |  | MIC range | category |  | MIC | category |  |  | MIC range | category |  | MIC | category | MIC | category |  | MIC range | category |  |
| CCUG 74415 | > 256.00 | R | 16.00 | R | R | >2 | R | Yes | 1.00 | S | R | I | 1.00 | S | Yes | 64.00 | R | 8.00 | R | R | >8 | R | Yes |
| CCUG 74416 | > 256.00 | R | 2.00 | R | S | >2 | R | No | < 0.25 | S | S | ≤1 | <=0.25 | S | Yes | 64.00 | R | 8.00 | R | R | >8 | R | Yes |
| CCUG 74417 | 8.00 | R | 32.00 | R | R | >1 | R | Yes | 4.00 | R | R | >2 | 4--8 | R | Yes | 32.00 | R | 16.00 | R | R | >8 | R | Yes |
| CCUG 74418 | > 256.00 | R | 8.00 | R | R | >2 | R | Yes | > 8 | R | R | >2 | >8 | R | Yes | 8.00 | R | 8.00 | R | R | 4.00 | R | Yes |
| CCUG 74419 | 8.00 | R | 16.00 | R | R | >2 | R | Yes | 4.00 | R | R | >2 | 4--8 | R | Yes | 32.00 | R | 16.00 | R | R | >8 | R | Yes |
| CCUG 74421 | 64.00 | R | 8.00 | R | R | >2 | R | Yes | 8.00 | R | R | >2 | 8.00 | R | Yes | 64.00 | R | 8.00 | R | R | >8 | R | Yes |
| CCUG 74422 | < 0.12 | S ≤ | 0.12 | S | S | 0.03-0.06 | S | Yes | < 0.25 | S | S | ≤1 | <=0.25 | S | Yes | 0.50 | S | 0.50 | S | S | 0.25-0.5 | S | Yes |
| CCUG 74423 | > 256.00 | R | 128.00 | R | R | >2 | R | Yes | 0.50 | S | S | ≤1 | 0.50 | S | Yes | 64.00 | R | 8.00 | R | S | >8 | R | No |
| CCUG 74424 | < 0.12 | S ≤ | 0.06 | S | S | <=0.03 | S | Yes | 8.00 | R | R | >2 | >2 | R | Yes | 4.00 | R | 8.00 | R | R | 4.00 | R | Yes |
| CCUG 74425 | < 0.12 | S ≤ | 0.06 | S | S | 0.03-0.06 | S | Yes | 8.00 | R | R | >2 | 8.00 | R | Yes | 0.50 | S | 0.50 | S | S | 0.25-0.5 | S | Yes |
| ATCC49619 | < 0.12 | S ≤ | 0.12 | S | S | ≤0.25 | S | Yes | < 0.25 | S | S | ≤1 | 0.12-1 | S | Yes | 1 | S | 0.50 | S | S | 0.06-0.5 | S | Yes |

Table S4: Minimum inhibitory concentration results for 129 isolates with MIC results, as determined by the phenotypic broth microdilution method and as predicted by the tools. Cells in blue were included in the EA calculation.

For the EA calculation, the same MIC range for the broth microdilution and the predictive tool were used. The values less than the lowest MIC from broth microdilution were taken together to correspond to the lowest end of the MICs from the predictive tool according to ISO 20776-1 guideline (29).

|  |  | penicillin | | | | | | | | | | | | | | | | | | | | | | | |
| --- | --- | --- | --- | --- | --- | --- | --- | --- | --- | --- | --- | --- | --- | --- | --- | --- | --- | --- | --- | --- | --- | --- | --- | --- | --- |
|  |  | AREScloud MIC (EA=88.4) | | | | | | | | | | | | Pathogenwatch MIC (EA=92.2) | | | | | | | | | | | |
|  |  | - | 0.03 | 0.062 | 0.125 | 0.25 | 0.5 | 1 | 2 | 4 | 8 | 16 | Total | - | <=0.03 | <=0.06 | 0.12 | 0.25 | 0.5 | 1 | 2 | 4 | 8 | 16 | Total |
| broth microdilution MIC | <=0.015 |  | 18 |  |  |  |  |  |  |  |  |  | 18 |  | 18 |  |  |  |  |  |  |  |  |  | 18 |
|  | 0.03 |  | 2 |  |  |  |  |  |  |  |  |  | 2 |  | 2 |  |  |  |  |  |  |  |  |  | 2 |
|  | 0.06 |  |  | 1 |  |  |  |  |  |  |  |  | 1 |  |  | 1 |  |  |  |  |  |  |  |  | 1 |
|  | 0.125 |  |  | 2 | 5 |  | 1 |  |  |  |  |  | 8 |  | 1 |  | 5 | 1 | 1 |  |  |  |  |  | 8 |
|  | 0.25 |  |  | 1 | 2 | 13 | 1 |  |  |  |  |  | 17 |  |  |  | 2 | 15 |  |  |  |  |  |  | 17 |
|  | 0.5 |  |  |  |  | 35 | 8 | 1 |  |  |  |  | 44 |  |  |  |  | 39 | 4 |  | 1 |  |  |  | 44 |
|  | 1 |  |  |  |  | 3 | 7 |  |  |  |  |  | 10 |  |  |  |  | 4 | 4 | 2 |  |  |  |  | 10 |
|  | 2 |  |  |  |  |  |  | 4 | 4 |  |  |  | 8 |  |  |  |  |  |  |  | 8 |  |  |  | 8 |
|  | 4 |  |  |  |  |  |  | 8 |  | 2 | 2 |  | 12 |  |  |  |  |  |  | 1 | 8 | 3 |  |  | 12 |
|  | 8 |  |  |  |  |  |  | 1 |  | 7 |  |  | 8 |  |  |  |  |  |  |  | 1 | 3 | 4 |  | 8 |
|  | 16 |  |  |  |  |  |  |  |  | 1 |  |  | 1 |  |  |  |  |  |  |  |  | 1 |  |  | 1 |
|  | Total | 0 | 20 | 4 | 7 | 51 | 17 | 14 | 4 | 10 | 2 | 0 | 129 | 0 | 21 | 1 | 7 | 59 | 9 | 3 | 18 | 7 | 4 | 0 | 129 |

|  |  | amoxicillin | | | | | | | | | | | | | | | | | | | | | | | |
| --- | --- | --- | --- | --- | --- | --- | --- | --- | --- | --- | --- | --- | --- | --- | --- | --- | --- | --- | --- | --- | --- | --- | --- | --- | --- |
|  |  | AREScloud MIC (EA=95.3) | | | | | | | | | | | | Pathogenwatch MIC (EA=96.1) | | | | | | | | | | | |
|  |  | - | 0.03 | 0.062 | 0.125 | 0.25 | 0.5 | 1 | 2 | 4 | 8 | 16 | Total | - | <=0.03 | <=0.06 | 0.12 | 0.25 | 0.5 | 1 | 2 | 4 | 8 | 16 | Total |
| broth microdilution MIC | <=0.015 |  | 8 |  |  |  |  |  |  |  |  |  | 8 |  | 8 |  |  |  |  |  |  |  |  |  | 8 |
|  | 0.03 |  | 12 |  | 2 |  |  |  |  |  |  |  | 14 |  | 12 | 2 |  |  |  |  |  |  |  |  | 14 |
|  | 0.06 |  |  | 2 | 24 |  |  |  |  |  |  |  | 26 |  | 1 | 24 | 1 |  |  |  |  |  |  |  | 26 |
|  | 0.125 |  |  | 3 | 23 | 3 |  |  |  |  |  |  | 29 |  |  | 18 | 9 | 2 |  |  |  |  |  |  | 29 |
|  | 0.25 |  |  |  | 10 | 3 |  |  |  |  |  |  | 13 |  |  |  | 9 | 4 |  |  |  |  |  |  | 13 |
|  | 0.5 |  |  |  | 1 | 2 | 2 |  | 1 |  |  |  | 6 |  |  |  | 1 | 3 | 1 | 1 |  |  |  |  | 6 |
|  | 1 |  |  |  | 1 |  |  | 7 |  |  |  |  | 8 |  |  |  | 1 | 1 |  | 5 | 1 |  |  |  | 8 |
|  | 2 |  |  |  |  |  |  | 9 | 2 |  |  |  | 11 |  |  |  |  |  |  | 11 |  |  |  |  | 11 |
|  | 4 |  |  |  |  |  |  | 1 | 2 |  |  |  | 3 |  |  |  |  |  |  | 2 | 1 |  |  |  | 3 |
|  | 8 |  |  |  |  |  |  |  |  |  | 6 |  | 6 |  |  |  |  |  |  |  |  |  | 6 |  | 6 |
|  | 16 |  |  |  |  |  |  |  |  |  | 5 |  | 5 |  |  |  |  |  |  |  |  |  | 2 | 3 | 5 |
|  | Total | 0 | 20 | 5 | 61 | 8 | 2 | 17 | 5 | 0 | 11 | 0 | 129 | 0 | 21 | 44 | 21 | 10 | 1 | 19 | 2 | 0 | 8 | 3 | 129 |

|  |  | cefotaxime | | | | | | | | | | | | | | | | | | | | | | | |
| --- | --- | --- | --- | --- | --- | --- | --- | --- | --- | --- | --- | --- | --- | --- | --- | --- | --- | --- | --- | --- | --- | --- | --- | --- | --- |
|  |  | AREScloud MIC (EA=97.7) | | | | | | | | | | | | **P**athogenwatch MIC (EA=98.4) | | | | | | | | | | | |
|  |  | - | - | 0.062 | 0.125 | 0.25 | 0.5 | 1 | 2 | 4 | 8 | 16 | Total | - | - | <=0.06 | 0.12 | 0.25 | 0.5 | 1 | 2 | 4 | 8 | 16 | Total |
| broth microdilution MIC | <=0.015 |  |  | 11 |  |  |  |  |  |  |  |  | 11 |  |  | 11 |  |  |  |  |  |  |  |  | 11 |
|  | 0.03 |  |  | 9 |  |  | 1 |  |  |  |  |  | 10 |  |  | 9 | 1 |  |  |  |  |  |  |  | 10 |
|  | 0.06 |  |  | 7 | 22 |  |  |  |  |  |  |  | 29 |  |  |  | 22 |  |  |  |  |  |  |  | 29 |
|  | 0.125 |  |  |  | 32 | 7 |  |  |  |  |  |  | 39 |  |  |  | 39 |  |  |  |  |  |  |  | 39 |
|  | 0.25 |  |  |  | 2 | 5 |  | 1 |  |  |  |  | 8 |  |  |  | 5 | 2 |  | 1 |  |  |  |  | 8 |
|  | 0.5 |  |  |  |  |  | 2 | 3 |  |  |  |  | 5 |  |  |  |  |  | 3 | 2 |  |  |  |  | 5 |
|  | 1 |  |  |  |  |  | 2 | 3 | 4 |  |  |  | 9 |  |  |  |  |  | 1 | 8 |  |  |  |  | 9 |
|  | 2 |  |  |  |  |  |  | 11 | 1 | 1 |  |  | 13 |  |  |  |  |  |  | 7 | 6 |  |  |  | 13 |
|  | 4 |  |  |  |  |  |  | 1 |  |  |  |  | 1 |  |  |  |  |  |  |  | 1 |  |  |  | 1 |
|  | 8 |  |  |  |  |  |  |  |  |  |  | 4 | 4 |  |  |  |  |  |  |  |  |  | 4 |  | 4 |
|  | 16 |  |  |  |  |  |  |  |  |  |  |  | 0 |  |  |  |  |  |  |  |  |  |  |  | 0 |
|  | Total | 0 | 0 | 27 | 56 | 12 | 5 | 19 | 5 | 1 | 0 | 4 | 129 | 0 | 0 | 27 | 67 | 2 | 4 | 18 | 7 | 0 | 4 | 0 | 129 |

Table S5.- Phenotypic categorical results versus categorical results of the predictive tools (AREScloud and PW) for each antibiotic included in the comparison. The blue boxes indicate the number of isolates with identical categorical result used to calculate categorical agreement.

|  |  | | **penicillin** | | | | | | | | | | | | |
| --- | --- | --- | --- | --- | --- | --- | --- | --- | --- | --- | --- | --- | --- | --- | --- |
|  |  | | **AREScloud** | | | | | | | | **Pathogenwatch** | | | | |
|  |  | | S | | I | | R | | Total | | S | | I | R | Total |
| **phenotypic** | S | | 391 | | 18 | | 0 | | 409 | | 391 | | 18 | 0 | 409 |
|  | I | | 3 | | 84 | | 0 | | 87 | | 1 | | 86 | 0 | 87 |
|  | R | | 0 | | 9 | | 12 | | 21 | | 0 | | 10 | 11 | 21 |
|  | Total | | 394 | | 111 | | 12 | | 517 | | 392 | | 114 | 11 | 517 |
|  |  | | **amoxicillin** | | | | | | | | | | | | |
|  |  | | **AREScloud** | | | | | | | | **Pathogenwatch** | | | | |
|  |  | | S | | I | | R | | Total | | S | | I | R | Total |
| **phenotypic** | S | | 481 | | 2 | | 1 | | 484 | | 482 | | 2 | 0 | 484 |
|  | I | | 1 | | 7 | | 0 | | 8 | | 2 | | 5 | 1 | 8 |
|  | R | | 0 | | 10 | | 15 | | 25 | | 0 | | 13 | 12 | 25 |
|  | Total | | 482 | | 19 | | 16 | | 517 | | 484 | | 20 | 13 | 517 |
|  | |  | | **cefotaxime** | | | | | | | | | | | |
|  | |  | | **AREScloud** | | | | | | | | **Pathogenwatch** | | | |
|  | |  | | S | | I | | R | | Total | S | | I | R | Total |
| **phenotypic** | | S | | 486 | | 4 | | 0 | | 490 | 486 | | 4 | 0 | 490 |
|  |  | I | | 2 | | 19 | | 1 | | 22 | 1 | | 21 | 0 | 22 |
|  |  | R | | 0 | | 1 | | 4 | | 5 | 0 | | 1 | 4 | 5 |
|  | | Total | | 488 | | 24 | | 5 | | 517 | 487 | | 26 | 4 | 517 |

|  |  | **erythromycin** | | | | | | |
| --- | --- | --- | --- | --- | --- | --- | --- | --- |
|  |  | **AREScloud** | | | | **Pathogenwatch** | | |
|  |  | S | I | R | Total | Non-R | R | Total |
| **phenotypic** | S | 401 | 2 | 2 | 405 | 404 | 1 | 405 |
|  | R | [16](applewebdata://17A88B1B-7A0B-4272-8FCC-0846C2070181#ERY_ARES_PW_VME_ME!A1) | 1 | 95 | 112 | 60 | 52 | 112 |
|  | Total | 417 | 3 | 97 | 517 | 464 | 53 | 517 |
|  |  | **trimethoprim-sulfamethoxazole** | | | | | | |
|  |  | **AREScloud** | | | **Pathogenwatch** | | | |
|  |  | S | R | Total | Non-R | I | R | Total |
| **phenotypic** | S | 365 | 35 | 400 | 365 | 30 | 5 | 400 |
|  | I | 3 | 35 | 38 | 2 | 4 | 32 | 38 |
|  | R | 2 | 77 | 79 | [1](applewebdata://8560C1A8-4F7B-48D0-9511-B4F88E8BCAB2#COTRI_ARES_VME!A1) | 27 | 51 | 79 |
|  | Total | 370 | 147 | 517 | 368 | 61 | 88 | 517 |
|  |  | **tetracycline** | | | | | | |
|  |  | **AREScloud** | | | | **Pathogenwatch** | | |
|  |  | S | I | R | Total | Non-R | R | Total |
| **phenotypic** | S | 393 | 1 | 8 | 402 | 396 | 6 | 402 |
|  | R | [22](applewebdata://3309CC2D-B3CF-41CF-8A20-663FCD3661CB#TETRA_ARES_VME!A1) | 1 | 92 | 115 | 54 | 61 | 115 |
|  | Total | 415 | 2 | 100 | 517 | 450 | 67 | 517 |

Table S6.- Measurement of the processing time for each tool to upload and process a batch of 20 sequences in 3 different times.

|  |  | Pathogenwatch | AREScloud |
| --- | --- | --- | --- |
| 1 | Uploading starting time (UTC+1) | 05:42 | 09:20 |
|  | Upploading completing time (UTC+1) | 06:20 | 09:58 |
|  | Results available time (UTC+1) | 07:50 | 11:25 |
|  | Total time to results (in hours) | 02:08 | 02:05 |
| 2 | Uploading starting time (UTC+1) | 13:32 | 11:20 |
|  | Upploading completing time (UTC+1) | 13:55 | 11:55 |
|  | Results available time (UTC+1) | 18:50 | 14:00 |
|  | Total time to results (in hours) | 05:18 | 02:40 |
| 3 | Uploading starting time (UTC+1) | 19:23 | 16:55 |
|  | Upploading completing time (UTC+1) | 20:00 | 17:33 |
|  | Results available time (UTC+1) | 21:15 | 19:45 |
|  | Total time to results (in hours) | 01:52 | 02:50 |
|  | Average time to result | 03:06 | 02:31 |

Supplementary materials and methods

The models for AREScloud were trained using extreme gradient boosting and elastic net regularized regression algorithms. Each algorithm was trained on a training matrix filtered to 500 K, 100 K, and 50 K most informative features. The resulting six models were then stacked together using out-of-fold predictions of inner 3-fold cross-validation to generate a stacking model (33). The performance of the models on separate validation data was used for selecting the final model.

**REFERENCES**

33. Lüftinger L, Májek P, Beisken S, Rattei T, Posch AE. 2021. Learning from limited data: towards best practice techniques for antimicrobial resistance prediction from whole genome sequencing data. Front Cell Infect Microbiol 11:610348. https://doi.org/10.3389/fcimb.2021.610348
